# Role of Transesophageal Echocardiography in the Surgical Treatment of Malignant Thrombosis of the Superior Vena Cava

**DOI:** 10.7759/cureus.97357

**Published:** 2025-11-20

**Authors:** Veronica Gagliardi, Giuseppe Gagliardi

**Affiliations:** 1 School of Medicine, University of Padua, Padua, ITA; 2 Department of Anesthesiology Intensive Care and Pain Unit, Azienda Unità Sanitaria Locale 5 Polesana, Rovigo, ITA

**Keywords:** advanced monitoring techniques, malignancy-related superior vena cava syndrome, multimodal imaging approach, transesophageal echocardiography (tee), ultrasound guidance

## Abstract

This is a case of malignant superior vena cava thrombosis successfully treated during open surgical treatment. A 70-year-old woman presented in the emergency department for edema of the head, neck, eyelids, and upper limbs and dyspnea. The CT scan and CT arteriography showed an obstruction in the venous drainage in the superior vena cava associated with a mediastinal mass, a histotype B malignant thymoma. Therefore, radical surgical resection with correction of the superior vena cava thrombosis was performed. Preoperative transesophageal echocardiography showed pericardial effusion on the inferior apical level with a thickened pericardium. A thrombus was completely occluding the superior vena cava lumen, projecting toward the superior-anterior side of the right atrium, directed toward the right appendage. Cardiopulmonary bypass cannulation was carefully placed using ultrasound guidance to not invade the thrombotic mass, avoiding further embolic events. The postoperative transesophageal echocardiography showed that the thrombus was effectively removed, and the stabilization of the superior vena cava wall was successful. The right appendage was free from thrombosis, similar to the superior vena cava, and pericardial effusion was absent. In this framework, advanced monitoring is fundamental, making neurological, respiratory, and hemodynamic assessments important. As we could not employ the Swan-Ganz catheter given the anatomic obstacle, the transesophageal echocardiographic assessment was mandatory both for hemodynamic monitoring and surgical guidance. It has a role in assessing patients with complete occlusion of the superior vena cava, severe refractory symptoms, and potential thrombosis of venous collaterals. With improved anesthesiology and perioperative advanced monitoring, surgical procedures can be performed safely and effectively in selected patients.

## Introduction

Thymoma is the most common primary anterior mediastinal tumor in adults, accounting for 20% of identified cases. The invasion of blood vessels is rare. To date, there is no accepted standard treatment for vascular invasive thymoma due to a paucity in the literature regarding causes and complications of superior vena cava syndrome secondary to malignant thrombosis [1]. However, in the rare incidence of an intravascular thymoma, radical resection of the primary tumor and replacement of the invaded large blood vessels with grafts could be effective [2,3].

In this framework, the earliest signs and symptoms can reflect a superior vena cava syndrome. The condition leads to increased venous pressure in the upper body, with edema of the head, neck, and upper limbs, often associated with cyanosis and distended subcutaneous vessels [4]. Moreover, edema of the periorbital region with proptosis and conjunctival coloring may be present. In rapidly progressive severe superior vena cava syndrome, the edema may involve the larynx or pharynx, with consequent cough, hoarseness, dyspnea, stridor, and dysphagia, but rarely with a significant impairment of the airway. Cerebral edema can also occur, manifesting as headache, confusion, nausea, dizziness, visual disturbances, and coma. Furthermore, reduced venous return may cause systemic hypotension, resulting in syncope [5].

Symptoms usually develop over two weeks in approximately one-third of patients, which might be longer in other cases. Before treating superior vena cava syndrome, if the patient is stable, a histologic diagnosis should be established [6]. Symptom relief can be achieved by supplying oxygen, elevating the head of the bed, and administering steroids. On the contrary, bending over, coughing, or assuming a supine position may exacerbate the symptoms [7,8].

## Case presentation

**Figure 1:**
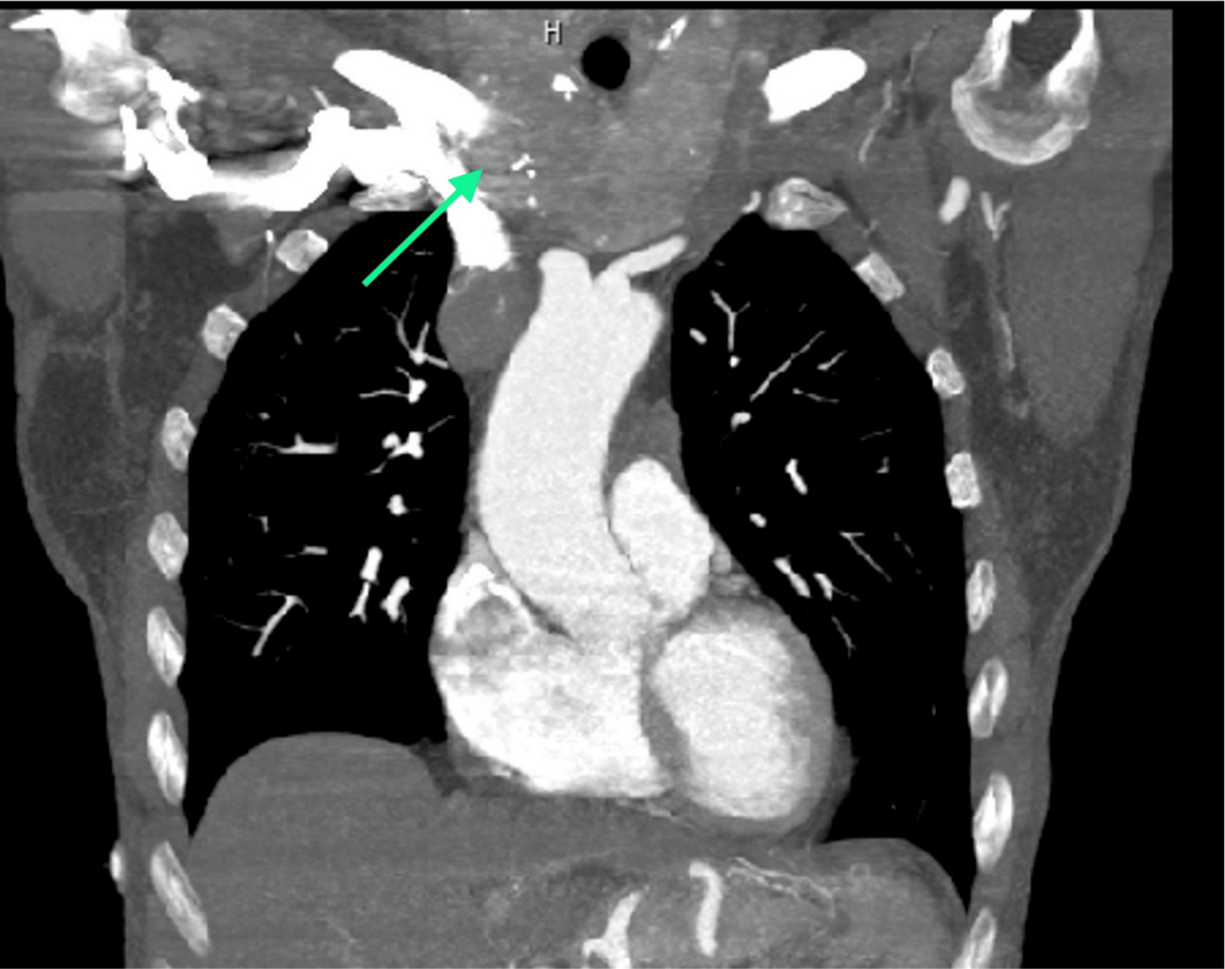
Coronal view of the mediastinum mass.

**Figure 2:**
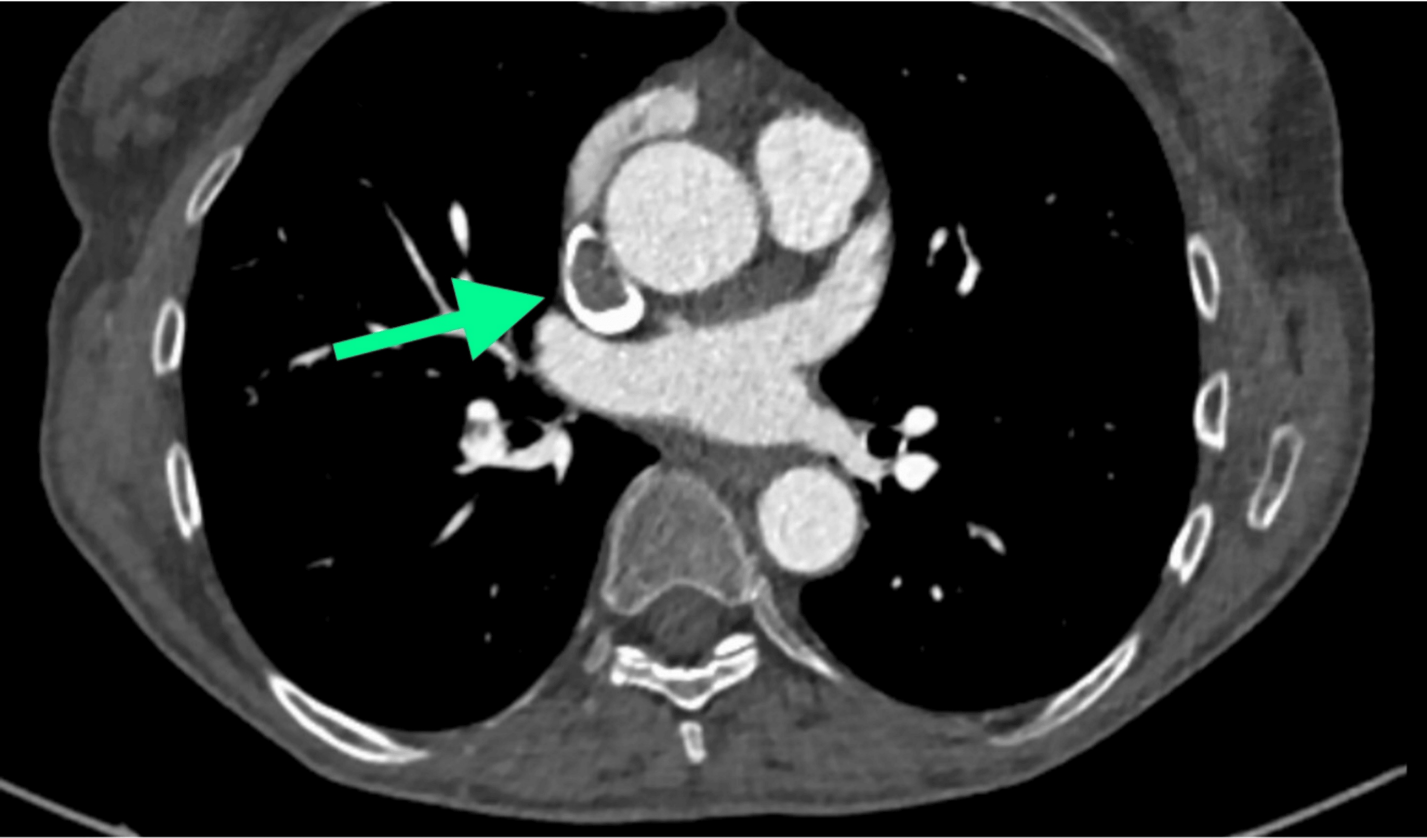
Axial view of the occlusion of the superior vena cava.

**Figure 3:**
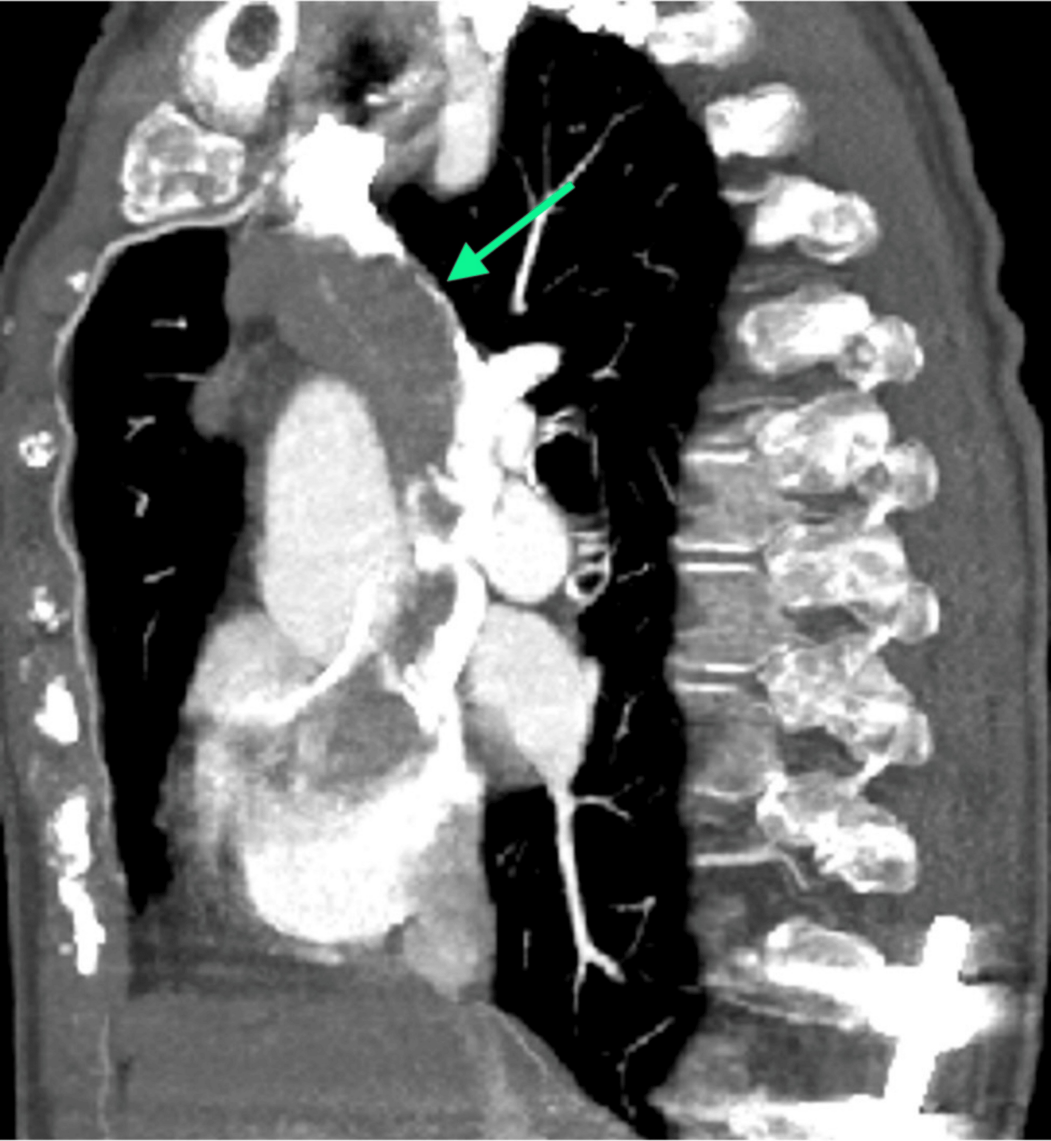
Sagittal view of the occlusion of the superior vena cava.

**Figure 4:**
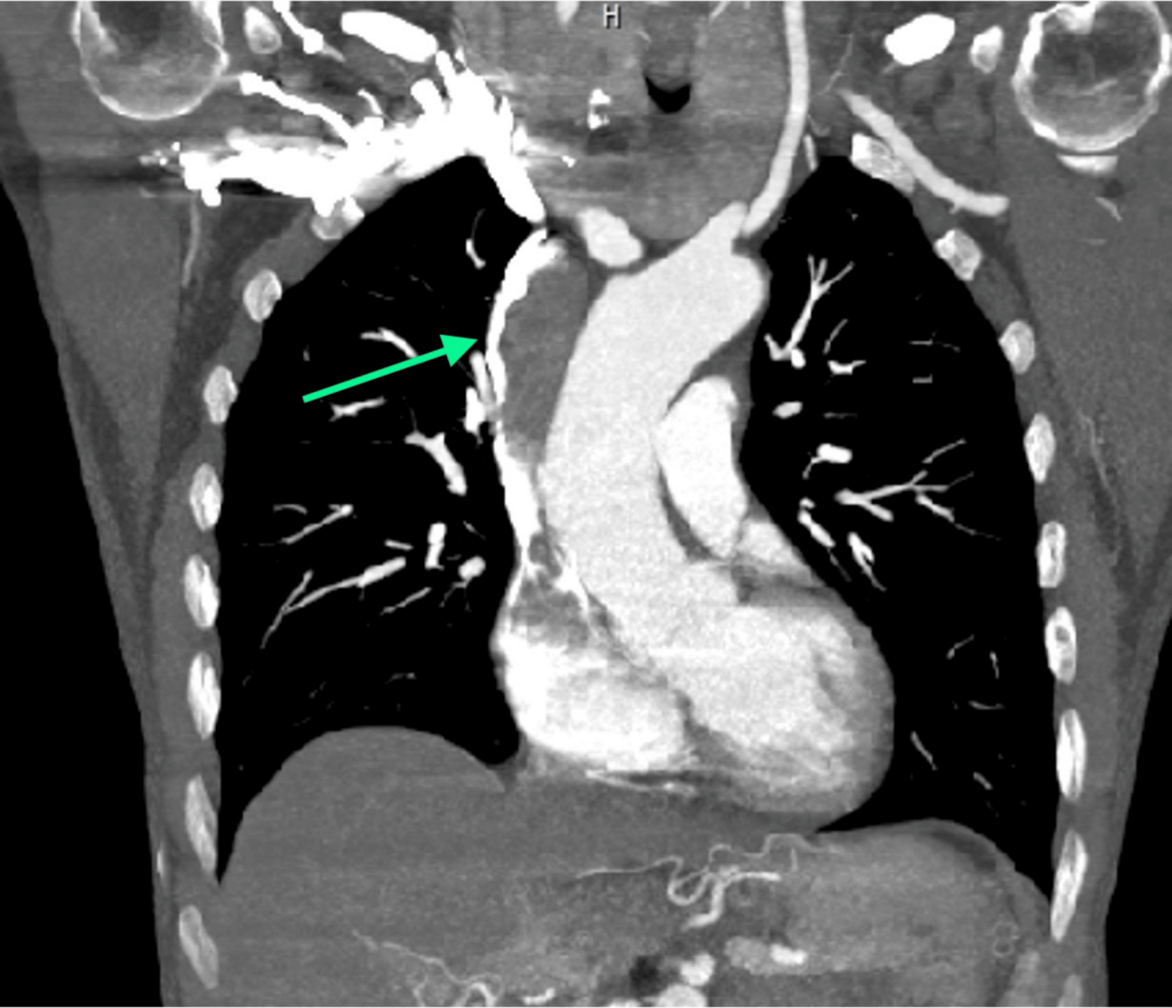
Coronal view of the occlusion of the superior vena cava.

A 70-year-old woman presented in the emergency department for edema of the head, neck, eyelids, and upper limbs, as well as dyspnea. CT imaging and CT arteriography demonstrated an obstruction in the venous drainage in the superior vena cava, which appeared to be due to an endovascular thrombosis, associated with a hypovascular, multilobed mass in the anterior-superior mediastinum, in the thymic region, with a major axis of approximately 40 × 25 mm and a craniocaudal extension of approximately 60 mm. More specifically, the mass infiltrated the confluence of the innominate veins, with endoluminal extension occupying the entire superior vena cava (craniocaudal extension of approximately 11 cm), approaching the right atrium appendage. There was also lymphadenopathy in the main hilar mediastinum. Moreover, signs of pulmonary embolism at the origin of the right interlobar artery were detected, but there were no significant alterations in the lung parenchyma and interstitium (Figures 1-4).

To provide a histopathological diagnosis, a surgical biopsy was performed, which revealed a histotype B malignant thymoma. Therefore, radical surgical resection was recommended. As the superior vena cava was infiltrated and occluded, a surgical correction was also performed.

In the operating theater, the patient arrived neurologically intact, with her pupils isocoric, isocyclic, and normophotoreactive. She had a moderate face, neck with eyelid edema, and mild cyanosis of the upper trunk. Hematologic values were normal; in particular, hemoglobin was 11 g/dL, platelets were 101,000/mm³, and fibrinogen was 218 mg/dL.

Non-invasive neuromonitoring was placed at the beginning of the operative procedure. It showed that the electroencephalographic trace was normal, and brain oxygenation was adequate. At first, adequate volume supplementation was satisfied before the induction of general anesthesia, and adequate blood pressure and peripheral perfusion were achieved. Central venous access was obtained via the left femoral vein.

Then, protective mechanical ventilation with low positive end-expiratory pressure levels was maintained to not increase the intrathoracic pressure, an element that could worsen the underlying condition. We employed the double-lumen tracheal tube to allow selective one-lung ventilation during the resection of the thymoma and the mediastinal lymph nodes. We did not need vasopressors or inotropes during this phase. Lactate was negative, and blood gases and pH were normal.

**Figure 5:**
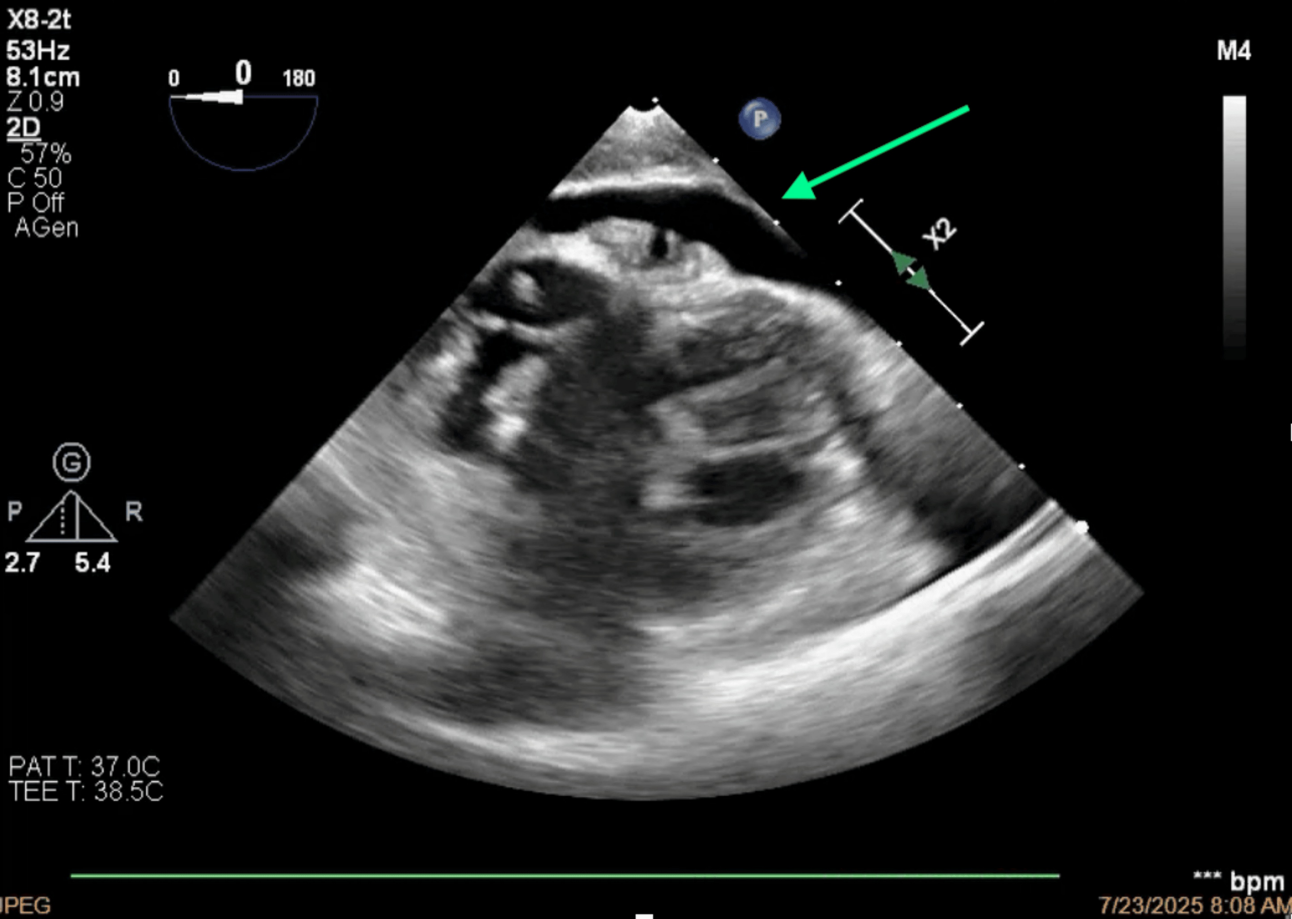
Transgastric mid-apical view showing pericardial effusion.

**Figure 6:**
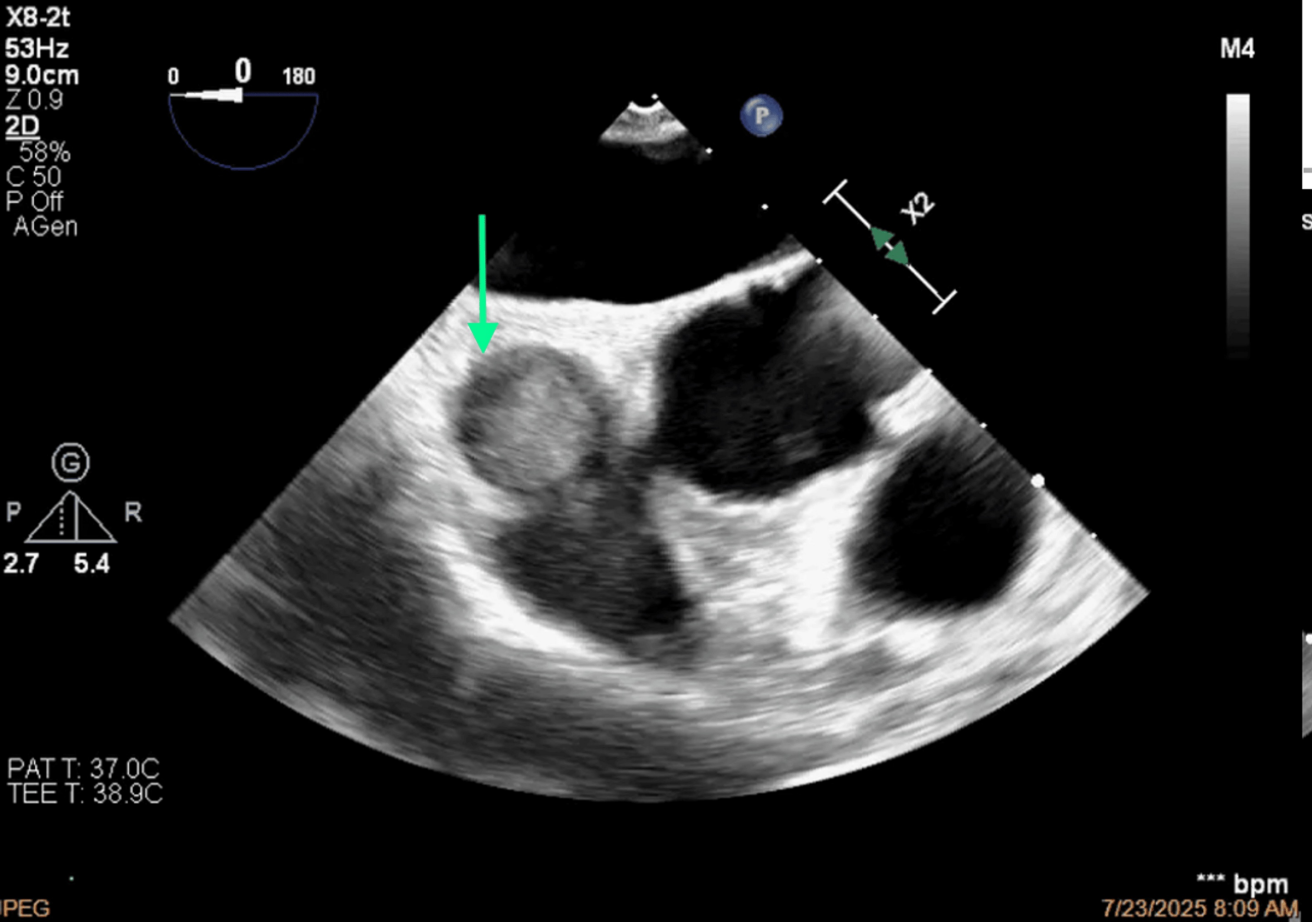
Thrombosis of the superior vena cava projecting toward the anterior-superior side of the right atrium.

**Figure 7:**
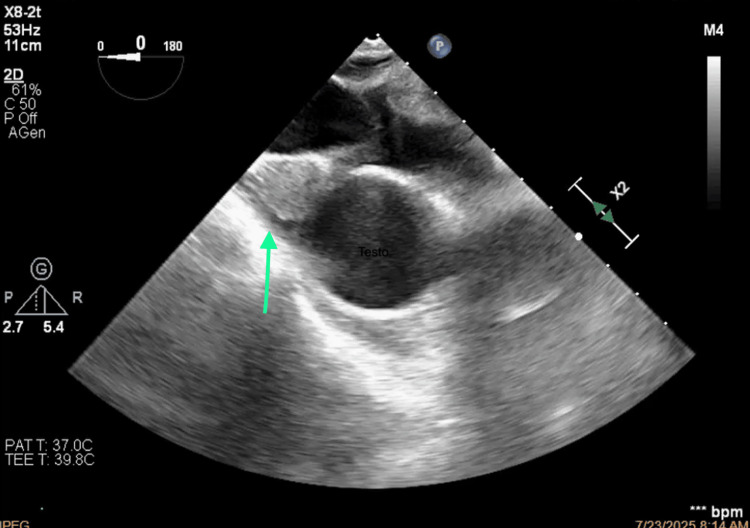
Short-axis view of the occlusion of the superior vena cava.

**Figure 8:**
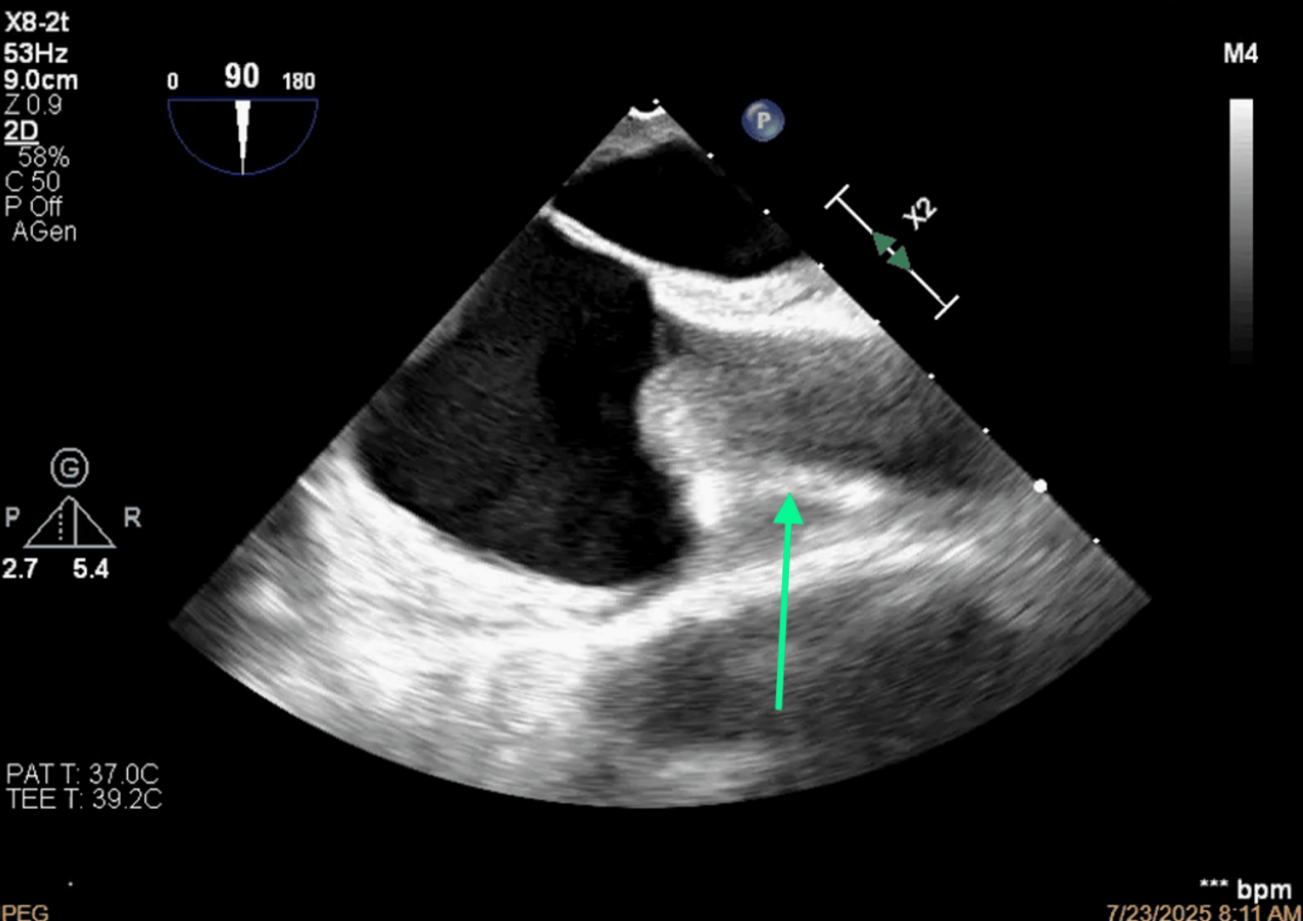
Bicaval view of the thrombosis of the superior vena cava.

**Figure 9:**
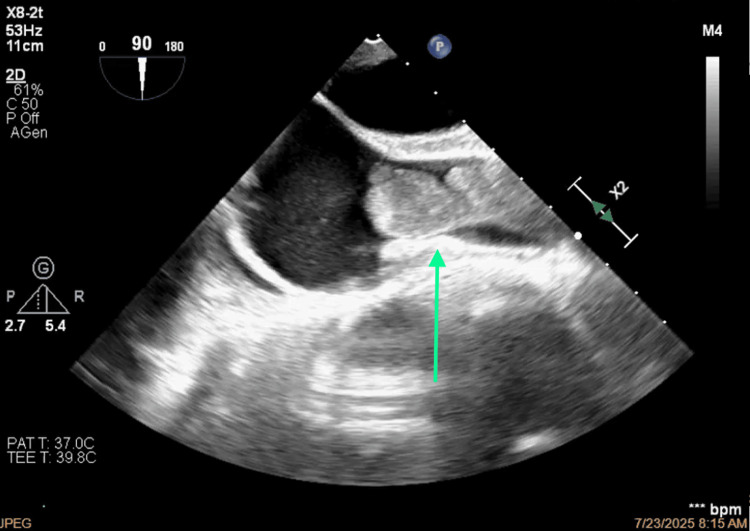
Morphology of the thrombus.

**Figure 10:**
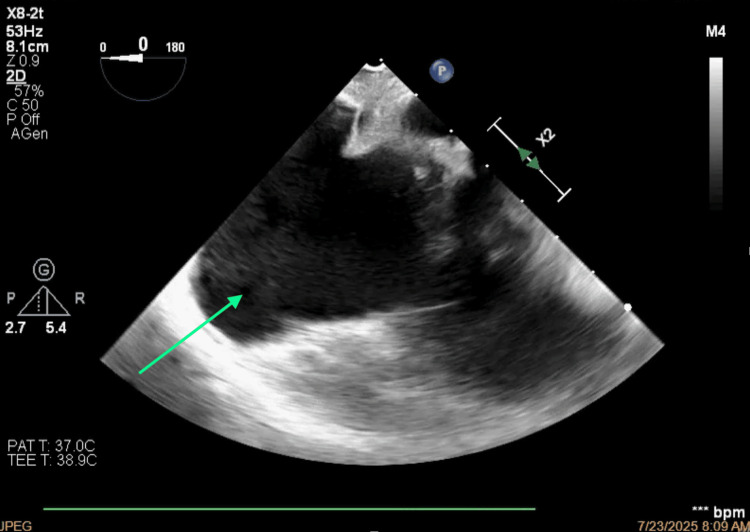
Right atrium free from thrombotic masses.

**Figure 11:**
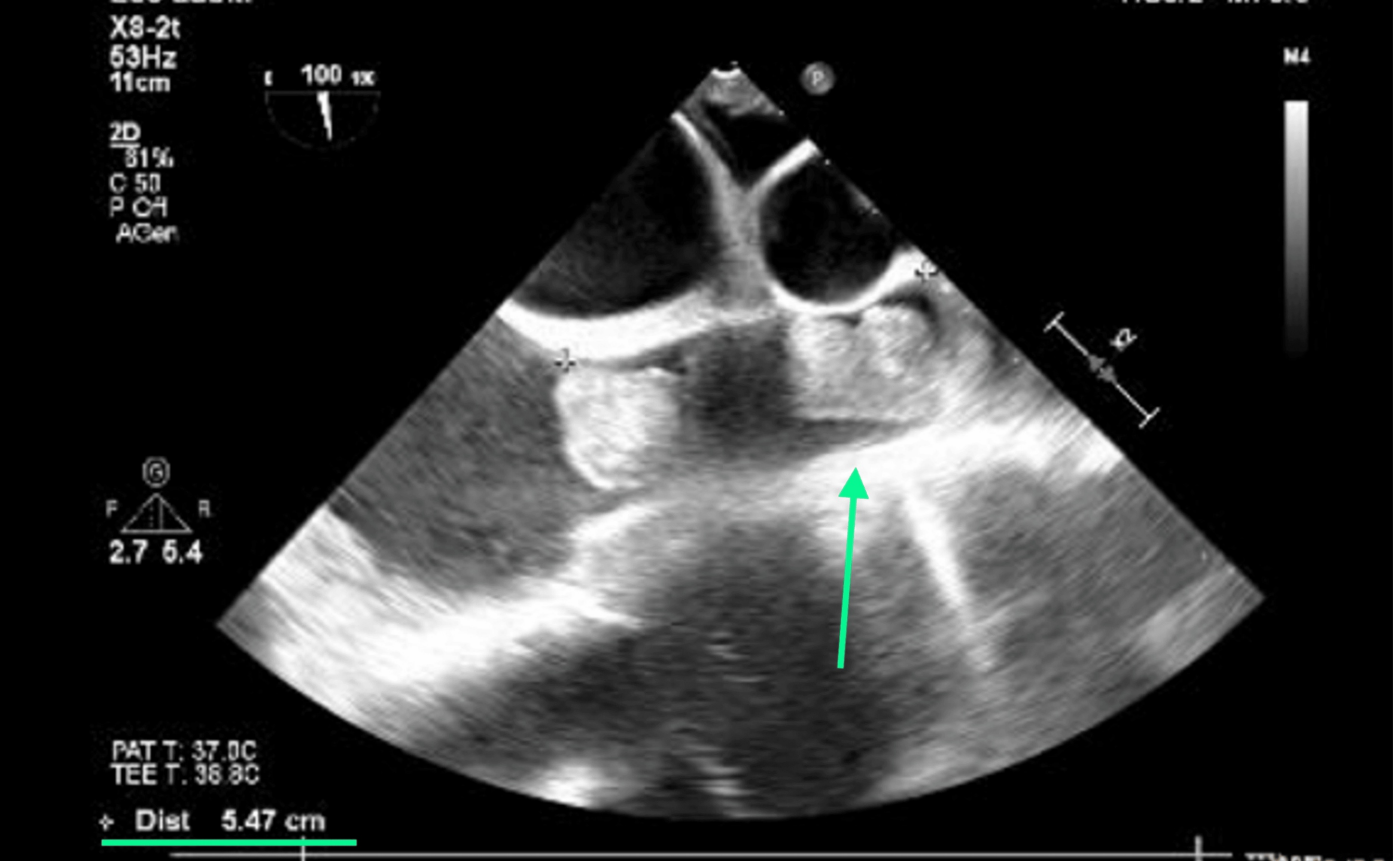
Extension of the thrombus in the superior vena cava, revealing complete obliteration until more than 5 cm.

The preoperative transesophageal echocardiography showed normal function of the left ventricle and the right ventricle, mild aortic regurgitation, mild tricuspid regurgitation, a well-functioning mitral valve, and normal bi-atrial dimensions. Pericardial effusion was detected at the inferior, medial-apical level, suggesting an invasion of the pericardium (Figure 5). A thrombus was completely occluding the superior vena cava lumen, projecting toward the superior-anterior side of the right atrium, and towards the right appendage. There were no signs of atrial communication (Figures 6-11).

**Figure 12:**
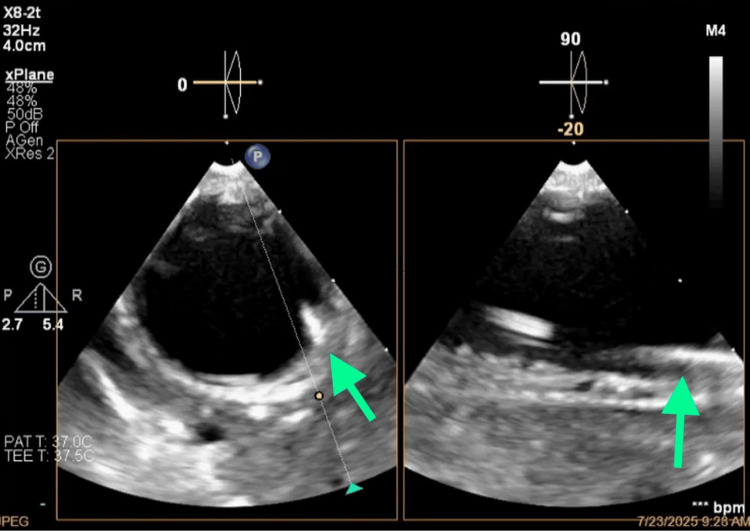
Ultrasound guidance of arterial cannulation.

**Figure 13:**
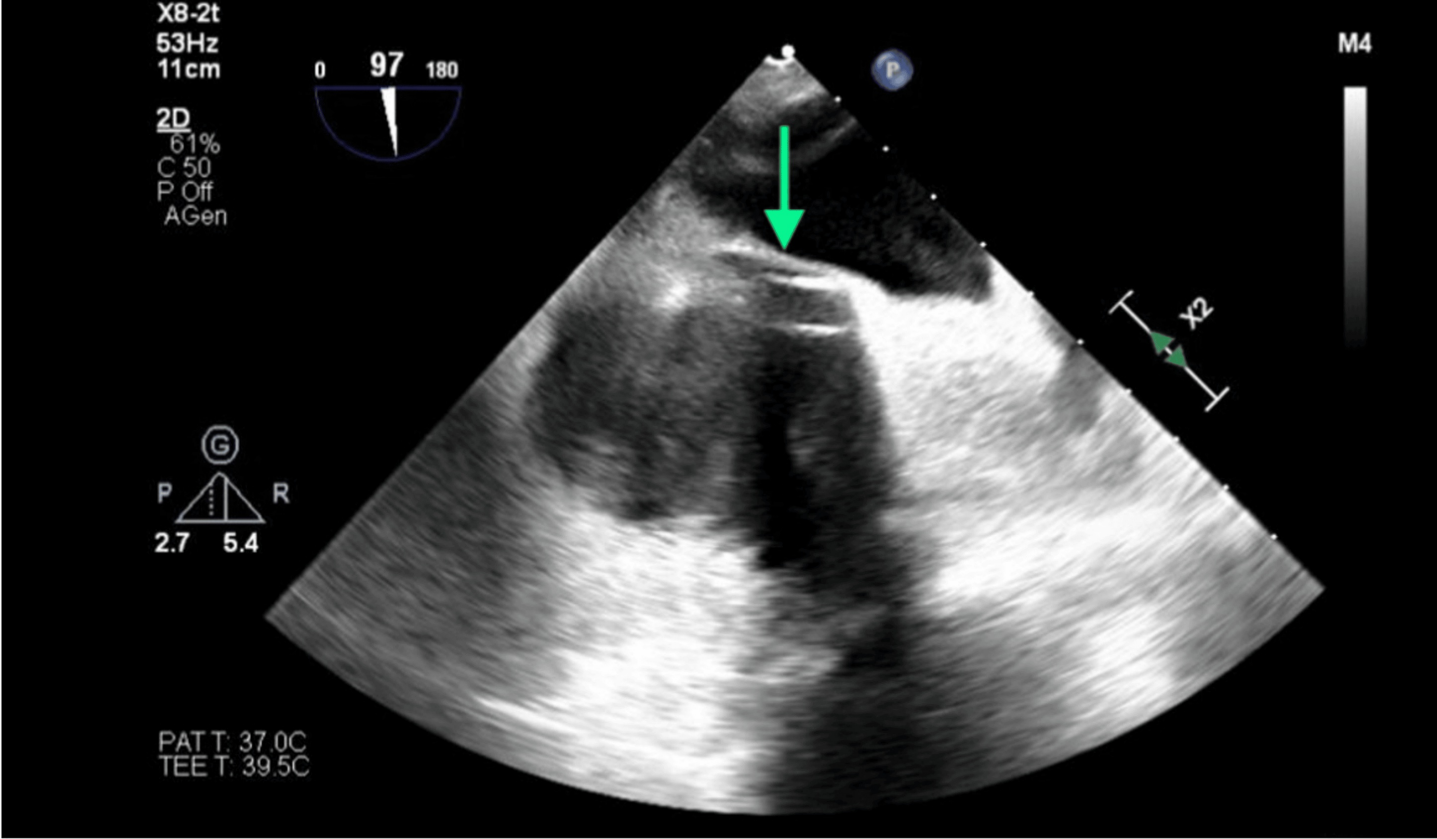
Ultrasound guidance of venous cannulation.

Surgically, cardiopulmonary bypass cannulation was carefully inserted using ultrasound guidance (Figures 12, 13) to disrupt the thrombotic mass, avoiding further embolic events. In this context, the drainage cannula involved only the inferior vena cava and was inserted in the right femoral vein, whereas the infusion cannula was inserted in the right femoral artery. We assumed that collateral circulation was present and could provide an adequate venous drainage, as the physiological assessed parameters were not severe, probably due to a chronic development of superior vena cava syndrome.

Nevertheless, during cardiopulmonary bypass, neurological monitoring detected a moderate reduction in brain oxygenation due to high levels of deoxygenated hemoglobin. Mannitol and thiopental were administered. Normoglycemia, normocapnia, and continued correction of electrolytes were maintained.

**Figure 14:**
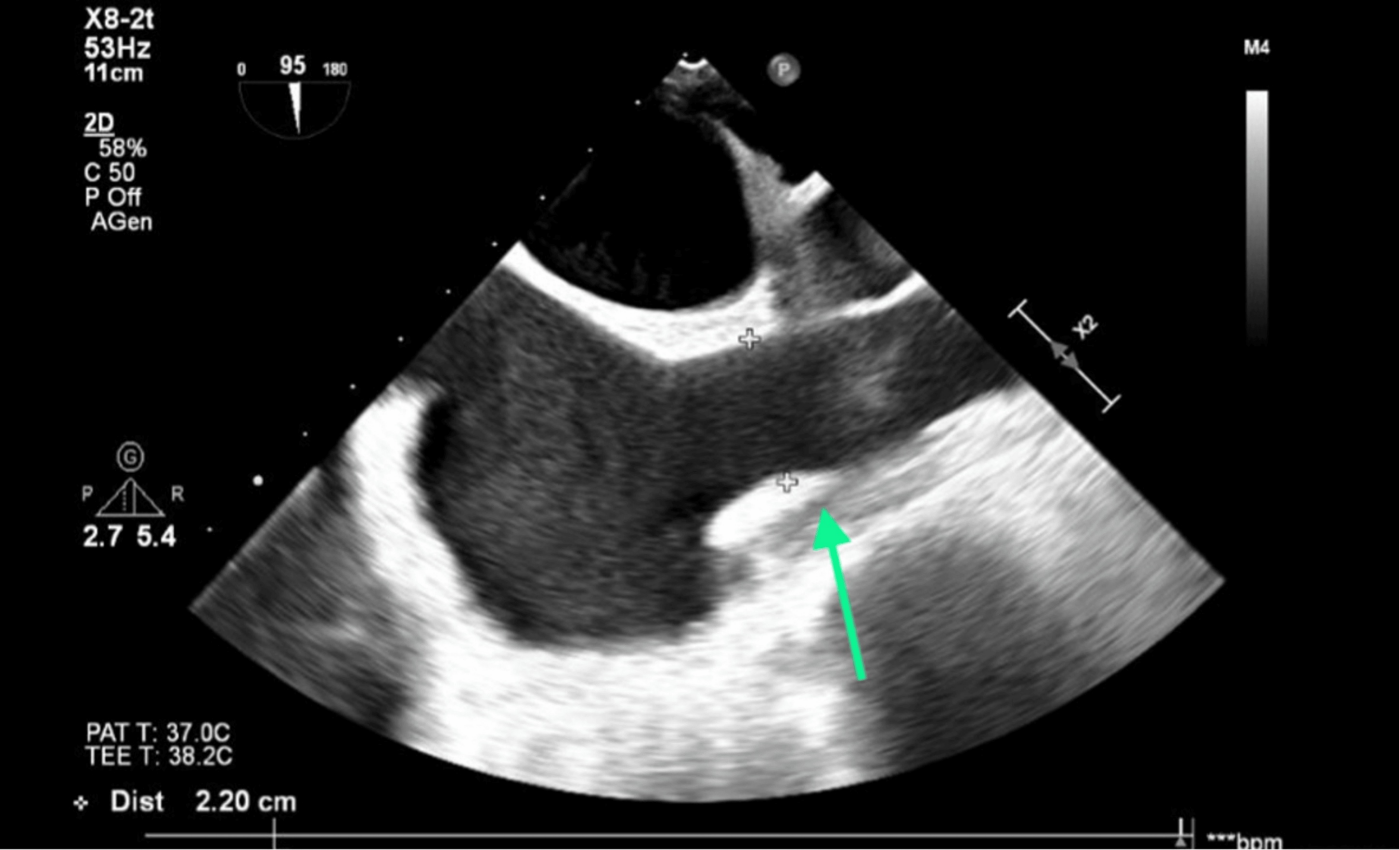
Reconstructed wall of the superior vena cava.

**Figure 15:**
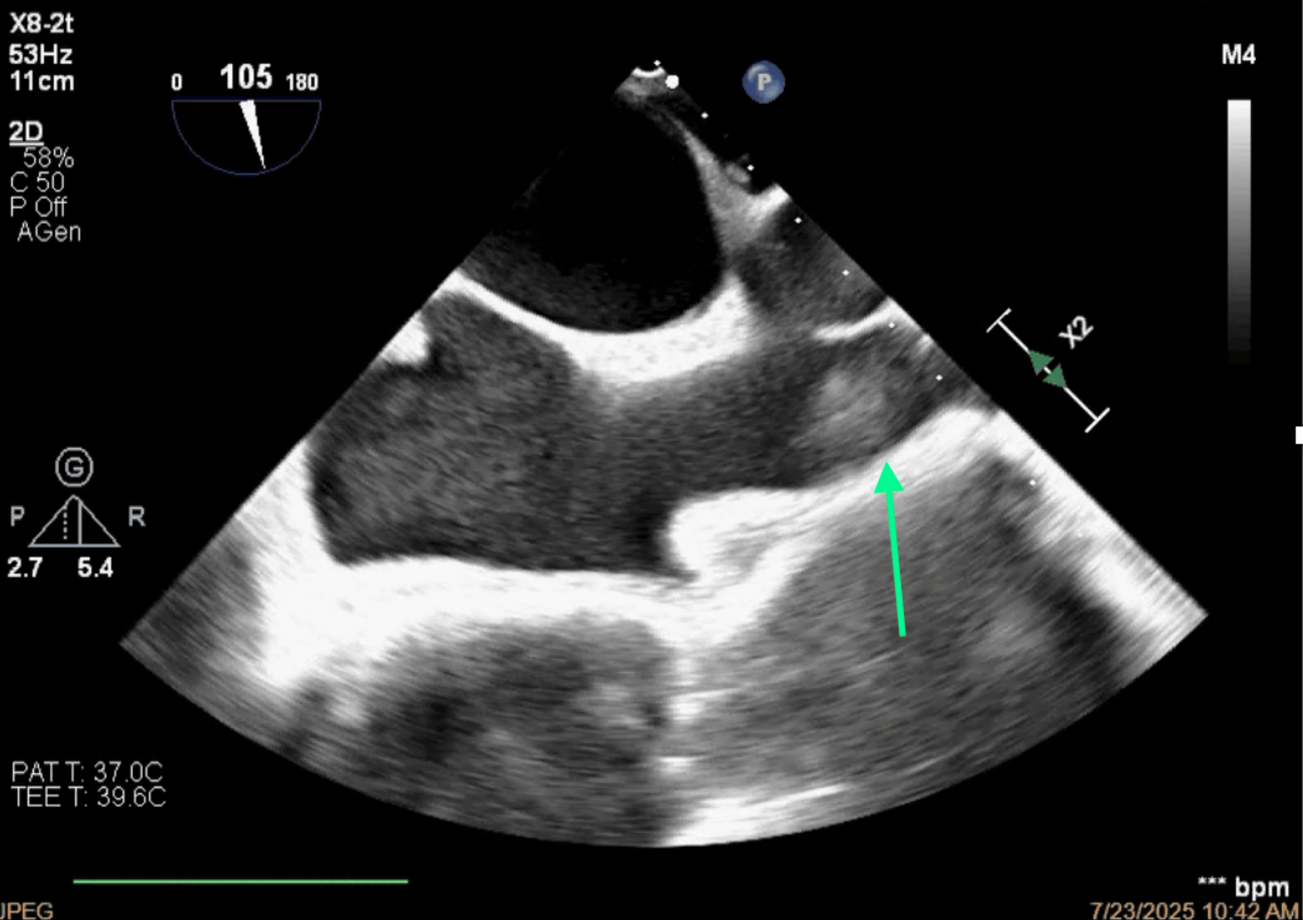
Complete removal of the thrombosis.

**Figure 16:**
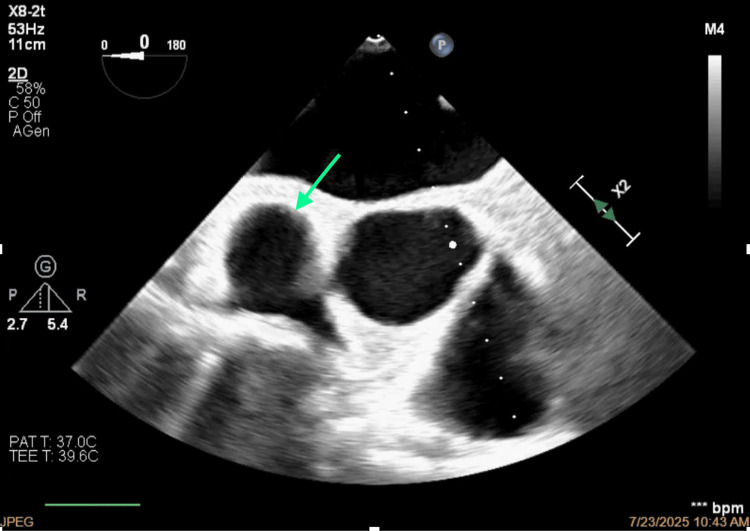
Short-axis view of the absence of the thrombosis in the superior vena cava.

**Figure 17:**
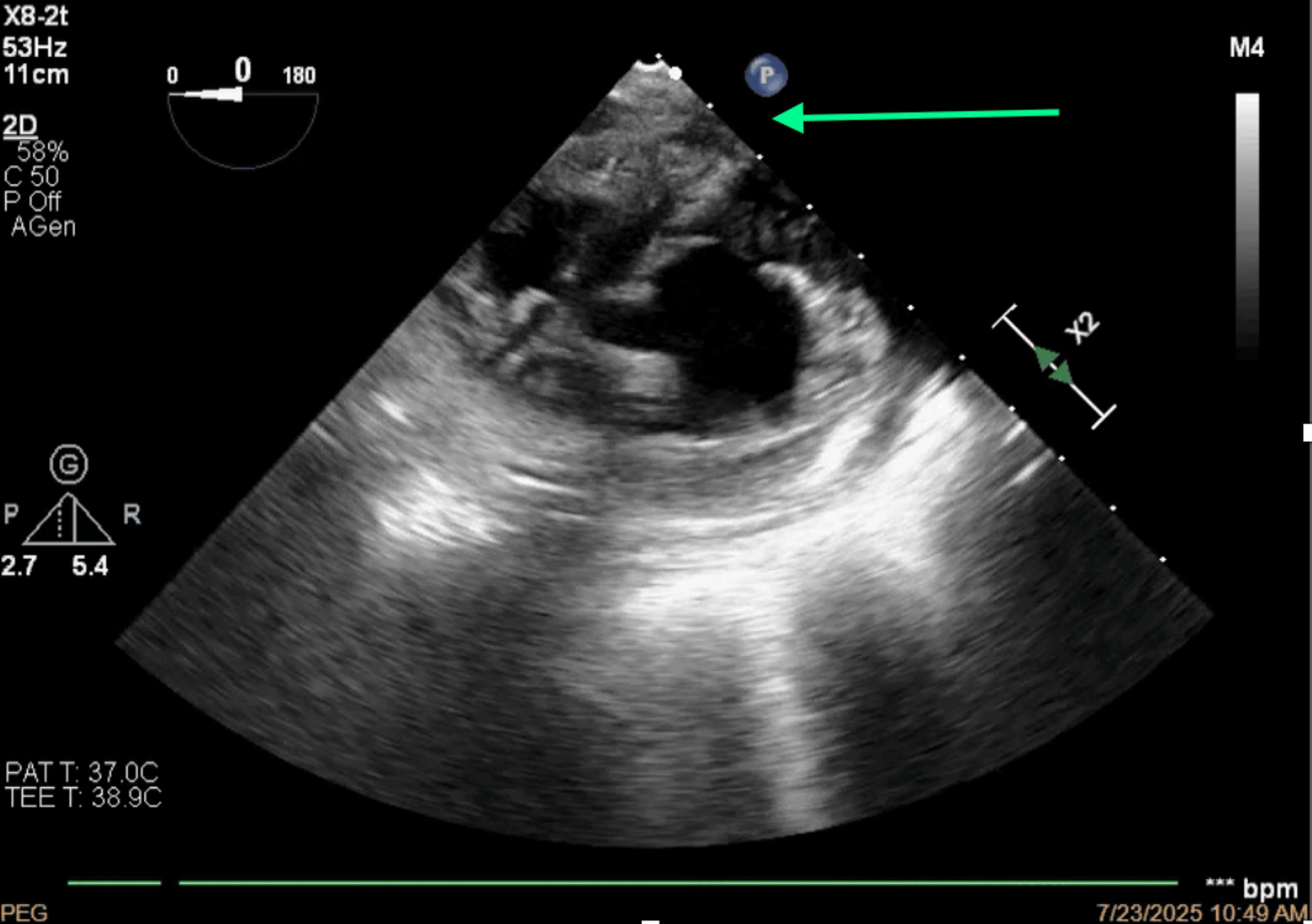
Transgastric mid-apical view revealing absence of pericardial effusion.

Weaning from the cardiopulmonary bypass was performed under ultrasound guidance, revealing normal physiological anatomy. The postoperative transesophageal echocardiography revealed that the thrombus had been effectively removed, and the reconstruction of the superior vena cava wall was successful (Figures 14, 15). The right appendage was free from thrombosis (Figure 16), as well as the superior vena cava, and no pericardial effusion was present (Figure 17).

The surgical procedure consisted of radical excision of the thymoma and the removal of the neoplastic thrombosis in the superior vena cava. As the malignant tumor infiltrated a wide region of the pericardium and part of the two pleurae, the pericardium was almost completely removed. The histological examination confirmed it was an intravascular infiltration of the thymoma with thrombus apposition, not a cancer-associated thrombosis. The superior vena cava was incised during the removal of the thrombus. The thrombus-neoplastic mass infiltrated the wall of the vessel, which involved complete removal with bovine pericardial patch reconstruction.

After the surgery, the patient was transferred to the intensive care unit, where she was weaned from sedation and mechanical ventilation and successfully extubated on the same day of surgery (postoperative day zero). Diuretic therapy was progressively introduced, including a loop diuretic and a potassium-sparing diuretic. She did not need vasopressor or inotropic support, and she did not manifest any signs of cardiac failure. She continued the anticoagulant therapy with low-molecular-weight heparin. Transthoracic echocardiographic assessment was performed every day, with confirmed preservation of bi-ventricular function, with no pericardial effusion. The patient was neurologically intact, without any peripheral or central injuries. After two days (postoperative day two), she was transferred to the surgical ward.

The case was discussed by the multidisciplinary oncologic team. Currently, the patient is undergoing chemotherapy, and a long-term follow-up has been planned.

## Discussion

In this framework, advanced monitoring is fundamental, including neurological, respiratory, and hemodynamic monitoring. As we could not employ the Swan-Ganz catheter given the anatomic obstacle, transesophageal echocardiography was mandatory both for hemodynamic monitoring and surgical guidance. There are few cases in the literature regarding the management of malignant thrombosis of the superior vena cava, and surgery is rarely indicated [9-11]. However, it offers prompt relief from the obstruction and the related symptoms. It has a role in treating patients with complete occlusion of the superior vena cava, extensive chronic thrombosis not anatomically suitable for endovascular treatment, severe refractory symptoms, and thrombosis of venous collaterals. With improved anesthesiology and perioperative advanced monitoring, surgical procedures can be performed safely and effectively in selected patients [11]. The intraoperative optimization of the parameters, such as blood pressure, brain oxygenation, and intrathoracic pressure, during positive pressure mechanical ventilation led to a successful outcome. In this context, transesophageal echocardiography is needed in the three main phases of the surgery. In the preoperative phase, it provided information about the global function of the heart, including the function of both ventricles, the atria, and the valves, as well as about the obstruction of the superior vena cava. In the intraoperative phase, it was fundamental in guiding the peripheral cannulation of the cardiopulmonary bypass and the weaning from it. In the postoperative phase, it confirmed the effective removal of the thrombosis and the repair of the wall of the vein. Moreover, after the surgery, it allowed the immediate assessment of the function of the heart, chambers, and valves, detecting if any iatrogenic injury occurred.

## Conclusions

This is a case of malignant superior vena cava thrombosis successfully treated with open surgery, associated with the resection of the thymoma to the removal of the completely occluding neoplastic and thrombotic mass in the superior vena cava. In this framework, intraoperative transesophageal echocardiography played a pivotal role, as it described and investigated the preoperative condition, the thrombus, its characteristics, and its position in the superior vena cava and in the right atrium and right atrium appendage. It was a fundamental guide for the cannulation, weaning from the cardiopulmonary bypass, confirmation of the complete removal of the thrombus, and correct reconstruction of the wall of the vessel.
